# Battle of the sexes: who is more variable, and does it really matter?

**DOI:** 10.1038/s41684-023-01164-7

**Published:** 2023-04-06

**Authors:** Bronwyn M. Graham

**Affiliations:** School of Psychology, University of New South Wales, Sydney, Australia

**Keywords:** Physiology, Biological techniques, Motivation

## Abstract

New research using high-dimensional behavioural analyses has further undermined the “females are the more variable sex” trope that often accompanies all-male studies. But when we consider the benefits to the inclusion of females in research, their lesser variability is only the icing on the cake. It’s just good science, really.

In a recent publication in *Current Biology*, Levy et al.^1^ used ‘motion sequencing’, an unsupervised machine learning algorithm, to obtain high-dimensional characterization of sub-components of behaviour (e.g., rearing, running) in adult male and female C57BL/6J mice during free exploration of an open-field arena over 15 consecutive days. The authors measured intra-individual variability (across repeated observations of a given mouse) and inter-individual variability (between different mice at a given observation). Both sexes exhibited large, stable, inter-individual differences, to the point where behaviour could be decoded to predict individual mouse identity with >80% accuracy. Equally noteworthy, both intra- and inter-variability were significantly greater in male mice than female mice.

Why is this important? Because years after the introduction of policies to equalize female and male representation in animal studies^2^, studies in males still outnumber those in females^3^. Moreover, despite multiple meta-analyses^3–6^ and individual studies^7^ demonstrating that data from females are not, as a rule, more variable than male counterparts, scientists *in this decade* are still stating that females are more variable than males as their major justification for all-male studies^3^. The Levy et al.^1^ study is another nail in the coffin of the “greater female variability” trope; and a mighty nail at that, because motion sequencing enabled nuanced, dynamic aspects of behaviour to be captured, affording high sensitivity. Their study is important, because when females are excluded from research on the basis of erroneous assumptions that have become so embedded, they persist not just within the scientific community, but also the broader public worldview^8^, we need convincing data to correct those assumptions.

So, females are not more (and sometimes, even less) variable than males; and now, what do we do with this information? The work by Levy et al.^1^, and others^3–7^, will only have impact if the *data are used*. To what end, and by whom? Ideally, by scientists contemplating whether or not to include females in studies. But also, by the gatekeepers of scientific credibility – the thousands of us who review manuscripts, funding submissions and institutional review board proposals, who by now, surely have sufficient empirical basis to reject justifications of female exclusion based on their perceived greater variability.

Sometimes resource constraints mean that only one sex can be studied. In these instances, Levy et al.^1^ suggest that females should be the default sex in studies of exploratory behaviour based on their lesser variability. But is greater variability alone *ever* a rational reason to exclude a sex (or any other group) from a study? Perhaps not. At some point, we seem to have conflated the desire for homogenous experimental effects with the need to investigate these effects in a highly homogenous population. Levy et al.^1^ clearly show that not only is homogeneity difficult to achieve, even in lab animals, but more importantly, we can rise to the challenge of studying heterogeneous populations, and accommodate their complexity using techniques like motion sequencing in combination with within-subjects designs. Moreover, citing lesser variability as the sole justification for selecting the “default” sex to study is particularly questionable. Sex is a complex construct that is meaningfully connected with the real-life phenomena that we aim to model in animal laboratory studies. Take animal models of health conditions. There are clear sex-based disparities in the prevalence and characteristics of many health conditions. Zucker and Beery^9^ demonstrated that in 2009, males were used more frequently than females even in studies of conditions that are more prevalent in females. A decade later, our own meta-analysis of animal research on fear, anxiety, and stress (conditions that are twice more prevalent in females relative to males) showed that more than two thirds of studies published in 2021 only included males^3^. The ‘male default’ persists, but thanks to Levy et al.^1^ and others^3–7^, this can no longer be justified on the basis of males being an easier (less variable) study subject. Is it right to now flip the argument on its head and position females as the new default, based only on their *lesser* inherent variability? The poetic justice is certainly pleasing. However, a more pertinent question when choosing between sexes (if the choice really must be made) might be “for which sex is this question most relevant”? If the answer to that question happens to be the more variable sex, then greater variance be damned.

When one comes around to the value of including females in non-human animal research, the question inevitably surfaces – “do I need to study the estrous cycle”? Again, Levy et al.’s^1^ data are thought-provoking. No behavioural sub-components predicted the estrous phase of an individual testing session. Behaviour only emerged as a modest predictor of estrous phase when averaged across each testing session from a given estrous phase. Moreover, inter-individual differences contributed to substantially more behavioural variance than estrous phase. It may be tempting to conclude that the estrous cycle can be safely disregarded in future studies of exploratory behaviour in mice. Levy et al.^1^ resist this temptation, perhaps out of consideration that a lack of difference in exploration across estrous phases does not necessarily signal identical neural mechanisms of exploration across estrous phases. Indeed, Levy et al.^1^ speculate that, given ovarian hormones modulate the neural circuits implicated in exploratory behaviour, there may be some mechanism that stabilizes behaviour across the estrous cycle. It therefore seems prudent to study the estrous cycle in subsequent studies seeking to identify the neural mechanisms of female exploratory behaviour. However, as when it comes to selecting the most appropriate sex to study, the answer to the broader question about studying the estrous cycle should always be “it depends on the question being asked”. The estrous cycle has clear relevance to female functioning and health, and in many instances, is an important variable to study^10^. But as with all variables, it will be more relevant to some questions than others. For instance, the estrous cycle may not be so relevant when exploring the mechanisms that account for Levy et al.’s^1^ observations of striking inter-individual variability in exploratory behaviour. Moreover, we must not forget that in addition to estrous cycling, there are many female-unique factors (such as pregnancy, menopause, and exposure to drugs like hormonal contraceptives) that warrant consideration^10^. Ultimately, all good science rests on thoughtful justification of experimental design and variable selection – good science in females is no exception.
